# Expression and Functional Characterization of NOD2 in Decidual Stromal Cells Isolated during the First Trimester of Pregnancy

**DOI:** 10.1371/journal.pone.0099612

**Published:** 2014-06-16

**Authors:** Yuan-yuan Zhang, Hui Chen, Chan Sun, Hua-zhao Wang, Mei-lan Liu, Yi- yang Li, Xiao-lu Nie, Mei- Rong Du, Da-jin Li, Jian-ping Zhang

**Affiliations:** 1 Department of Obstetrics and Gynecology, Sun Yat-sen Memorial Hospital of Sun Yat-sen University, Guangzhou, China; 2 Key Laboratory of Malignant Tumor Gene Regulation and Target Therapy of Guangdong Higher Education Institutes, Sun Yat-sen University, Guangzhou, China; 3 Laboratory for Reproductive Immunology, Hospital and Institute of Obstetrics and Gynecology, Fudan University Shanghai Medical College, Shanghai, China; 4 Department of Obstetrics and Gynecology, The first Hospital of JiLin University, Changchun, China; Queen's University, Canada

## Abstract

NOD2, one of the cytosolic proteins that contain a nuclear oligomerization domain (NOD), is a pattern recognition receptor (PRR) involved in innate immune responses to intracellular pathogens. Little is known, however, about the effect of NOD2 expression on the maternal–fetal relationship. Our aim was to elucidate the functions of NOD2 in normal decidual stromal cells (DSCs) from the first trimester. Tissues and DSCs were isolated from 26 patients with normal pregnancies that required abortion. The expression of NOD2 in deciduas/decidual stromal cells (DSCs) was examined by real-time PCR, immunohistochemistry, and In-cell western. DSCs containing NOD2 were stimulated by its ligand, muramyl dipeptide (MDP). The secretion of various cytokines and chemokines were measured by ELISA and the apoptotic rate was determined by flow cytometry. Treatment with MDP significantly elevated the expression of both NOD2 mRNA and protein levels in DSCs. In addition, MDP activation of NOD2 significantly increased IL-1β and MCP-1 cytokine expression in a dose dependent manner but had no effect on IL-12 expression. IL-1β and TNF-α also significantly increased the expression of NOD2 in DSCs, suggesting a positive feedback loop mechanism. Moreover, MDP stimulation augmented DSC apoptosis. In summary, the results suggest that NOD2 expression in DSCs plays an important role in protecting the embryo and preventing infection in the maternal-fetal interface.

## Introduction

Throughout pregnancy, the maternal immune response remains tolerant while still protecting it from infection from early implantation of the semi-allogeneic trophoblast to the full development of the fetus. An intrauterine infection is a significant threat to the pregnancy outcome. Clinical studies have shown a correlation between intrauterine infections and certain pregnancy complications, such as preterm labor and preeclampsia [1]–[3]. Therefore, protection of the maternal–fetal interface from infectious pathogens is critical during pregnancy.

The tissues in the maternal-fetal interface, including the decidua and placenta, have been shown to play an important role in the immune system during pregnancy [4], [5]. However, the mechanisms for maternal recognition of pathogens in the placental and decidual regions are still not fully elucidated. During early pregnancy, the decidua contains a large number of immune competent cells, namely natural killer (NK) cells, macrophages, dendritic cells and T cells. The majority of decidual immune cells belong to the innate immune system [6]–[8]. They express pattern recognition receptors (PRRs) that recognize pathogen-associated molecular patterns (PAMPs), allowing them to detect infection and activate the innate immune system [9]–[11].

To date, two major types of PRR have been identified. They are membrane bound PRRs, including toll-like receptors (TLRs), and intracellular PRRs, including nuclear oligomerization domains (NODs). The NOD receptors, NOD1 and NOD2, are mainly expressed in monocytes, macrophages, dendritic cells and epithelial cells [12]–[15] and are involved in the recognition of intracellular pathogens. NOD1 recognizes meso-diaminopimelic acid (iE-DAP) on Gram-negative bacteria while NOD2 recognizes muramyl dipeptide (MDP) that exists in both Gram-negative and Gram-positive bacteria [16]–[18]. Recognition and binding of NOD1 or NOD2 to their ligands induces a conformational change that triggers a downstream signaling cascade, resulting in the generation of an inflammatory response and the production of cytokines and chemokines [19], [20].

Previous studies have suggested an immunological role of NOD2 in the female reproductive tract. Lipopolysacchride (LPS) increases the expression of Nod2 in murine trophoblasts via TLR4 [21]. NOD2 proteins are expressed by first trimester trophoblasts [22], by the stroma during the late secretory phase of the menstrual cycle, and by decidualized stromal cells and the glandular epithelium from the first trimester [23]. MDP stimulation of endometrial endothelial cells significantly increased IL-8 and TNF-α mRNA, indicating the presence of functional NOD2. Abnormal expression of NOD2 is associated with various immune-related disorders and infectious diseases, such as Crohn's disease [24], asthma [25], preeclampsia and HELLP Syndrome [26].

In addition to decidual immune cells, non-immune decidual stromal cells (DSCs), which comprise 75% of decidual tissue, have also been shown to possess the potential for immunologic protection of the fetus at the maternal-fetal interface [27]. DSCs express the mRNA of ten TLRs, which are involved in the recognition of microbial antigens and the expression of TLR-2 and TLR-4 proteins [28], [29]. Recently, a report by King et al. showed that DSCs obtained from first trimester deciduas also expressed the cytosolic PRR protein, NOD2 [23]. These studies suggest that DSCs play a role in the immunologic protection at the maternal-fetal interface. However, a detailed understanding of NOD2 function in DSCs is still unknown. The aim of this study was to investigate the expression levels and function of NOD2 in DSCs during early pregnancy. Our study provides insight into the protective mechanisms at the maternal-fetal interface.

## Methods and Materials

### Participants

Twenty-six patients with a median age of 26 who had no history of pregnancy loss but required an induced abortion were recruited during their first trimester at Red-House hospital of Fudan University or SunYat-Sen Memorial Hospital of Sun Yat-Sen university January 21^st^ 2012 to October 6^th^ 2012. The inclusion criteria for URSA group were abortions occurring between 6–8 week of gestation, and normal genetics, anatomy, endocrine, hormone, certain coagulation and serum levels of immune regulatory proteins. The patients with infection, smoking and alcohol consumption, environmental factors, psychological trauma, and stressful life event were excluded. The surgical termination of pregnancies on those patients was performed at Red-House hospital of Fudan University or SunYat-Sen Memorial Hospital of Sun Yat-Sen university. All patients included in the study had signed consent forms before the tissue collection and the use of patient samples was approved by Human Research Ethics Committee of Obstetrics and Gynecology Hospital, Fudan University and Sun Yat-Sen University's Human Investigations Committee.

### Tissue collection

A viable intrauterine pregnancy and gestational age were confirmed by ultrasound scan and menstruation cycle. Some decidual tissues were fixed in 4% paraformaldehyde for 24 hrs, embedded, and stored in paraffin. Decidual stromal cells were isolated from fresh tissue which collected in ice-cold DMEM/F12 (Gibco, Grand Island, NY, USA). The tissues were transported to the laboratory within 30 min after the surgery, and washed in calcium- and magnesium-free Hanks balanced salt solution (HBSS).

### Reagents

Lipopolysaccharide (LPS) isolated from Escherichia coli (0111:B4) was purchased from Sigma (St Louis, MO, USA). A specific NOD2 agonist, MDP, was purchased from Invitrogen (San Diego, CA, USA). Cytokines (Peprotech, Rocky Hill, USA) including IL-1β (Catalog 200-01B), IFN-γ (Catalog 300-02), IL-17A (Catalog 200-17), TNF-α (Catalog 300-01A) were used for stimulating the cells.

### Cell culture

Desidual stromal cells (DSCs) from decidual tissue from individual patients were dissociated by collagenase IV/DNase-I digestion (Sigma, Saint Louis, Missouri, USA) and isolated by discontinuous Percoll gradient centrifugation, as described[30]. DSCs of density between 1.042 and 1.062 g/ml were recovered and cultured in DMEM/F12 (Gibco, Grand Island, NY, USA) complete medium supplemented with 10% heat-inactivated fetal bovine serum (FBS, Gibco), 100 U/ml penicillin, and 100 µg/ml streptomycin in 5% CO_2_ at 37°C. After primary culture for 30 min at 37°C in 5% CO_2_, non-adherent hematopoietic cells were washed away, leaving 98% pure DSCs, as confirmed by staining of mouse anti- Cytokeratin7 mAb and mouse anti-vimentin mAb (ZSGB-BIO, Beijing, China).

### RNA extraction from decidual cells and real-time PCR

The isolated DSCs were seeded at a density of 5×10^5^ cells/well in 12-well plates. After 12 h incubation in DMEM/F12 with 10% FBS, cells were treated with or without LPS (Sigma, St Louis, MO, USA) or MDP for 72 hrs or with cytokines (IL-1β, TNF-α, IFN-γ, IL-17A) for 24 hrs at the indicated concentrations. Cells were harvested and total RNA was extracted using trizol (Invitrogen, Carlsbad, CA, USA) protocol. Total cellular RNA (1 µg) was reverse transcribed into cDNA by using the cDNA Synthesis Kit (Fermentas, Waltham, USA) with oligo (dT)18 in a Mastercycler personal PCR machine (Eppendorf AG, Hamburg, Germany).

Real time-PCR mixtures contained SYBR Premix ExTaq II (2×) (Takara, Dalian, China), Forward and reverse primers for NOD2 or forward and reverse primers for GAPDH (all Applied Sangon Biotech, Shanghai, China); 3 µl cDNA was diluted into 12 µl, and 2 µl dilution was added for each PCR. PCRs were run on an ABI 7900HT (Perkin-Elmer Applied Biosystems, USA). Primer pairs for the gene amplification for NOD2 were as follows: 5′-TGCGGACTCTACTCTTTGAGC-3′ (forward) and 5′-CCGTGAACCTGAACTTGAACT-3′ (reverse); for human glyceraldehyde-3-phosphatedehydrogenase (GAPDH): 5′-GCACCGTCAAGGCTGAGAAC-3′ (forward) and 5′-TGGTGAAGACGCCAGTGGA-3′ (reverse). The reactions were carried out in 95°C for 30 sec, followed by 35 cycles at 95°C for 3 sec and 60°C for 30 sec. NOD2 and GAPDH expression were analyzed in parallel in all samples. Melting curve data were collected to check the RT-PCR specificity. Each cDNA was amplified in triplicate and the corresponding sample without reverse transcriptase (ddH_2_O) was included as the negative control. The expression of the NOD2 was normalized to that of GAPDH. The replicates were then averaged, and fold induction was determined in a ΔΔCt-based fold-change calculations.

### In-cell Western

We used an established assay called in-cell Western to determine the protein level of NOD2 in the DSCs, as described by Egorina et al. [31] with the following modifications. After freshly isolated DSCs (1.5×10^4^/well, 96-well plate) were starved in DMEM/F12 supplemented with 1% FBS for 12 hrs, these cells were incubated with or without cytokines (IL-1β, TNF-α, IFN-γ or IL-17A at concentrations of 1 ng/ml, 10 ng/ml and 100 ng/ml) for 24 hrs, or LPS or MDP (0.1 µg/ml, 1 µg/ml and 10 µg/ml) for 72 hrs. Cells were immediately fixed with 4% paraformaldehyde for 20 min at room temperature. After washing with 0.1% Triton three times, cells were blocked by adding 100 µl of LI-COR Odyssey Blocking Buffer (LI-COR Biosciences, Lincoln, Nebraska, USA) for 90 min at room temperature. Cells were incubated overnight at 4°C with mouse anti-human mAb NOD2 (25 µg/ml, Abcam, Cambridge, UK) primary antibody and the rabbit anti-human β-actin (1∶80, Santa Cruz, CA, USA) which detected the control reference protein. Cells were washed three times with 1×PBS, and incubated in the dark with the corresponding secondary antibodies: anti-mouse IRDyeTM700DX conjugated (1∶60, affinity purified, Red fluorescence) and anti-rabbit IRDyeTM800DX conjugated (1∶80, affinity purified, green fluorescence), as recommended by the manufacturers (Rockland, Inc, Gilbertsville, PA, USA). Images of NOD2 were obtained using the Odyssey Infrared Imaging System (LI-COR Biosciences GmbH). The protein level of NOD2 was calculated as the ratio of the intensity of NOD2 (green fluorescence) to that of β-actin (red fluorescence). Each experiment was repeated three times and the average of three measurements was used as the result.

### Cytokine Quantitation in the Supernatant by ELISA

To detect cytokine secretion in DSCs, the DSCs (1×10^6^/well) were cultured with or without LPS or MDP for 72 h in 6-well flat bottom plates (Nunc, Roskilde, Denmark). The levels of cytokines (IL-1β, IL-4, IL-10, IL-12, TNF-α, IFN-γ, CCL-2/MCP-1 and CXCL12/SDF-α) in the supernatants were quantified using Valukine or Quantikine kit (R&D Systems, Minneapolis, MN, USA) following the manufacturer's instructions. The lowest limits of detection of the cytokine ELISA kits were described as the following: IL-1β, 3.9 pg/ml; IL-4, 31.2 pg/ml; IL-10, 7.8 pg/ml; IL-12, 7.8 pg/ml; IFN-γ, 15.6 pg/ml; and TNF-α, 15.6 pg/ml; CCL-2/MCP-1, 31.2 pg/ml; and CXCL12/SDF-α, 15.6 pg/ml.

### Immunohistochemistry

Immunohistochemical analysis was performed as previously described [22]. In brief, the tissue sections were then deparaffinized, rehydrated in descending grades of alcohol, and subjected to antigen retrieval by microwaving for 15 min using citric acid antigen repair fluid (Beyotime, ShangHai, China). NOD2 expression was detected by using the AB complex (streptavidin/peroxidase) method (ZymedHistostain plus Kit, Zymed, USA) following the manufacturer's instructions. Sections were incubated with mouse anti-human CK7 mAb (Santa Cruz, Heidelberg, Germany), mouse anti-human vimentin mAb (Santa Cruz, Heidelberg, Germany), mouse anti-human NOD2 mAb (Abcam, Cambridge, UK) or PBS as control overnight at 4°C. Tissue sections were stained with haematoxylin (Sigma), then dehydrated with ethanol and mounted from xylene.

### Apoptosis detection with Flow Cytometry

To detect the influence of NOD2 on apoptosis and the signal pathway, DSCs (2×10^5^/well) were cultured in 24-well flat-bottom plates (Nunc, Penfield, NY, USA) for 48 hrs with or without MDP. The attached DSCs were harvested by using 0.25% typsin (without EDTA). The DSCs in suspension (individual Falcon 2054 polystyrene round-bottom tubes, Becton Dickinson, Franklin Lakes, NJ, USA) were washed with DMEM/F12 medium, resuspended in 200 µl DMEM/F12 (without FBS) using the Annexin V-Biotin Apoptosis Detection Kit (Calbiochem, Merck KGaA, Germany). In parallel, isotypic IgG antibodies were used as controls. Samples were analyzed by FACS Calibur flowcytometer (Becton Dickinson) using Cell Quest software (BectonDickinson). Statistical analysis was conducted by using isotype matched controls as the reference.

### Statistics

All values were shown as the mean±SEM. Student *t*-test was performed to detect the significant difference of mRNA and protein expression of NOD2 and the secretion of cytokine levels and apoptosis rate in the DSCs between pretreatment and control groups. The differences were accepted as significant at *P*<0.05.

## Results

### NOD2 expression in the DSCs at the first trimester

**Figure 1:**
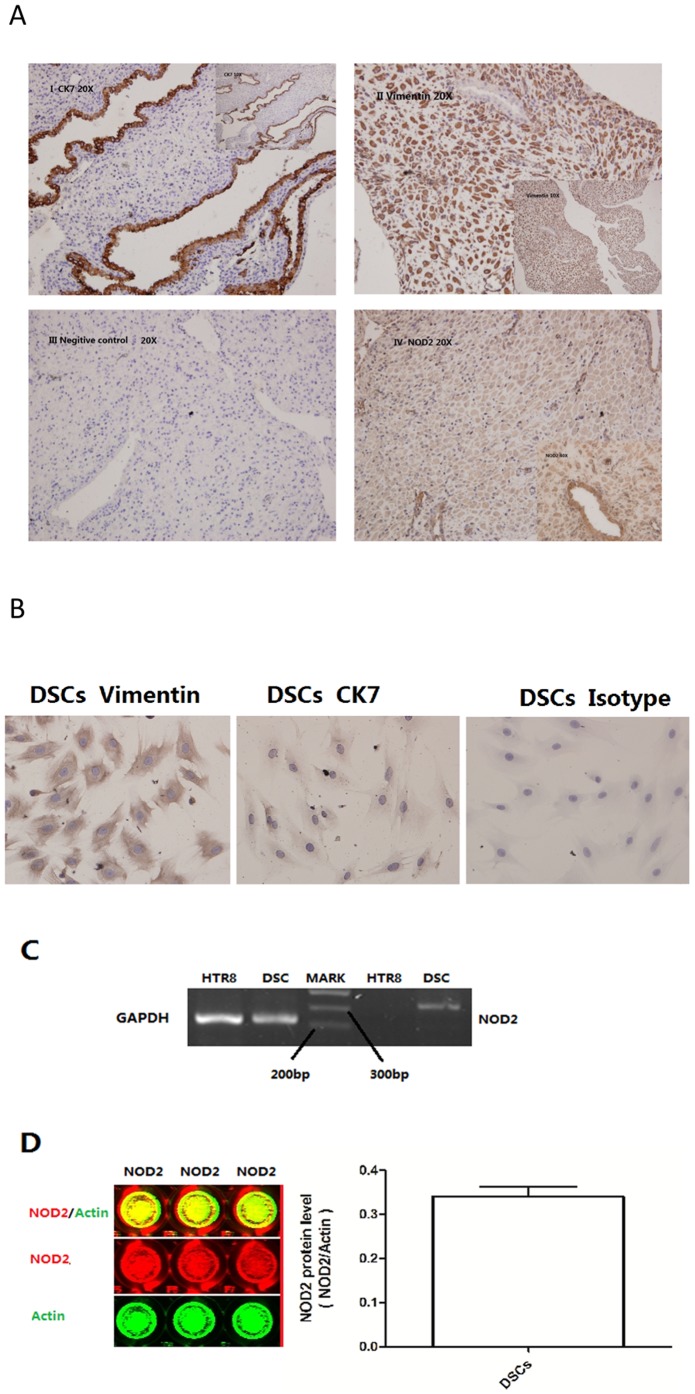
NOD2 expression in decidua and DSCs during the first trimester. (A) Immunolocalization of NOD2 decidua (40× magnification). DSCs were positively stained for vimentin but not CK7 in the cytoplasm (II), while decidual glandular epithelial cells were positive for CK7 and negative for vimentin (I). NOD2 staining in the deciduas and its pronounced localization to the decidual glandular epithelial cells was observed (IV). Negative controls (III) showed no staining. (B) Staining with vimentin but not CK7 confirmed the primary culture of DSCs. NOD2 expression in DSC primary culture was confirmed by RT-PCR (C) and in-cell Western blot analysis (D). The trophoblast cell line, HTR-8, was used as a negative control for RT-PCR.

To investigate whether NOD2 was expressed in the decidua from early pregnancy, immunohistochemical analysis was employed. As shown in Fig. 1A, DSCs were stained with vimentin but not CK7 in the cytoplasm, while decidual glandular epithelial cells were positive for CK7 and negative for vimentin. Positive immunoreactivity for NOD2 was observed in DSCs and decidual glandular epithelial cells and the signal appeared stronger in the latter (Fig.1A panels I, II and IV). To confirm this observation, we cultured primary cells from decidua and identified the primary DSCs by CK7 and vimentin staining (Fig. 1B). The expression of NOD2 in DSCs was evaluated by RT-PCR and in-cell Western blot analysis; both NOD2 mRNA and protein was detected in DSCs (Fig. 1C and 1D).

### The expression of NOD2 in DSCs was increased after stimulation with MDP and LPS

**Figure 2:**
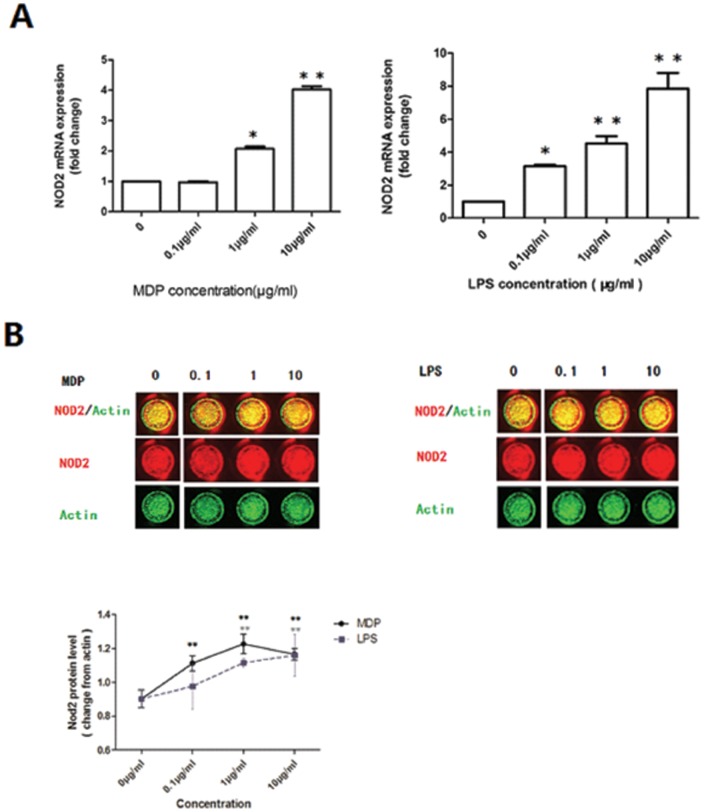
Effect of MDP and LPS on the expression of NOD2 mRNA and protein in normal DSCs. Data expressed as the mean of fold change ±SEM. Error bars depict the standard error of the mean, compared to the concentration of 0 µg/ml as a vehicle control. * P<0.05,* * P<0.01. (A) NOD2 gene expression is increased in DSCs after 72 hrs stimulation with MDP or LPS at different concentrations (0.1 µg/ml, 1 µg/ml, 10 µg/ml) (B) The protein levels of NOD2 was determined by In-cell Western. The figure represented the ratio of the intensity of NOD2 protein to that of β-actin. It was found that the protein level of NOD2 increased after treatment with MDP or LPS. The pictures were taken of representative wells of one of three individual experiments.

To investigate the effect of bacterial exposure on NOD2 expression and thus NOD2's ability to detect the invasion of pathogens during normal pregnancy, we stimulated the DSCs from normal pregnant women at first trimester with the NOD2 ligand MDP. The bacterial antigen LPS, which has been shown to activate NOD2 mRNA expression through TLR4 in trophoblast cells [21], was included as a control. Treatment of first-trimester normal DSCs with MDP or LPS significantly increased their mRNA and protein levels of NOD2 in a dose-dependent manner (Fig. 2).

### Chemokines and Cytokine Response to inflammatory stimuli of normal DSCs

**Figure 3:**
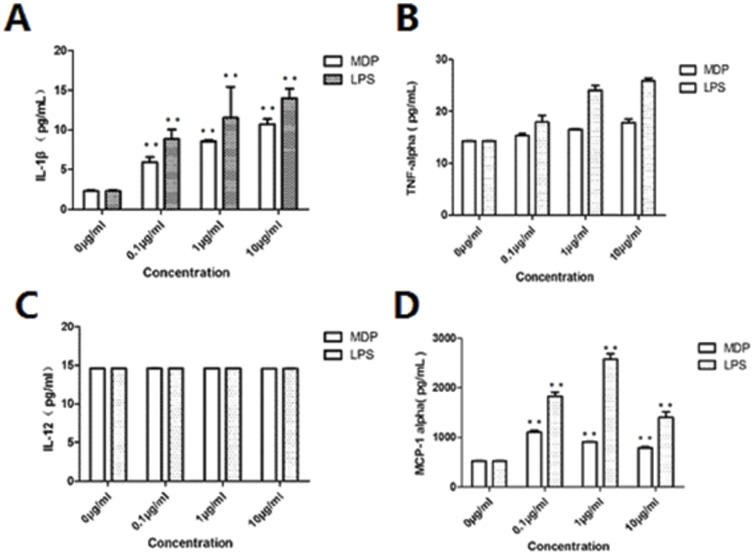
Effect of MDP and LPS on cytokine production by first trimester normal DSCs. The DSCs were treated with either control medium (0 µg/ml), MDP (0.1 µg/ml, 1 µg/ml, 10 µg/ml), LPS (0.1 µg/ml, 1 µg/ml, 10 µg/ml) for 72 hrs. Cell-free supernatants were collected and assayed for cytokine concentrations. The bar charts for (A) IL-1β, (B) TNF-α, (C) IL-12, (D) MCP-1 alpha (diluted by 5 times), show significant changes in cytokine levels compared to the control (*P<0.05; **P<0.01). Data were from one representative of four independent experiments.

We next examined the effect of NOD2 stimulation or LPS stimulation of DSCs on secretion of pro-inflammatory and anti-inflammatory cytokines and chemokines (IL-1β, IL-4, IL-10, IL-12, TNF-α, IFN-γ, CCL-2/MCP-1 and CXCL12/SDF-α). Stimulation of NOD2-expressing DSCs to MDP or LPS significantly increased the secretion of IL-1β, and MCP-1 (P<0.01; Fig. 3A, D), but not TNF-α or IL-12 (Fig. 3B, C). Although MDP and LPS stimulation of DSCs did not induce a significant change in TNF-α secretion (Figure 3B) compared that of the control, a dose dependent trend was clear. Moreover, we did not see significant changes in IL-4, IL-10, IFN-γ, and CXCL12/SDF-1α secretion after the stimulation (data not shown).

### Inflammatory cytokines increased the expression of NOD2

**Figure 4:**
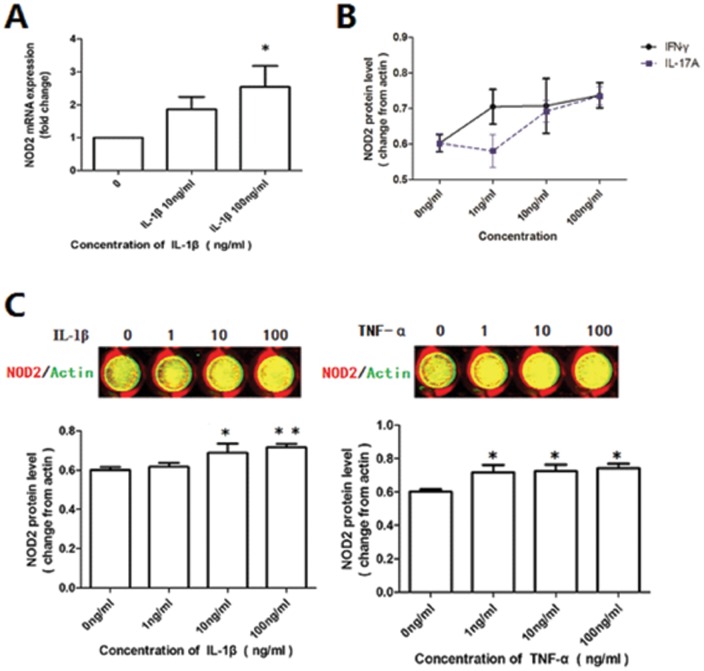
Effect of cytokines on the expression of NOD2 in first trimester DSCs. The primary-cultured DSCs from normal pregnant women were starved with 1% FBS for 12 h and then treated with vehicle, IL-1β, TNF-α, IFN-γ or IL-17A of different concentration (1 ng/ml, 10 ng/ml, 100 ng/ml) for 24 hrs respectively. The mRNA and protein level of NOD2 was determined by real-time PCR and In-cell Western. The pictures were from one representative of three individual experiments. (A) IL-1β increased the NOD2 mRNA expression in DSCs. (B) IFN-γ or IL-17A, but did not affect NOD2 expression after 24 hrs stimulation. (C) NOD2 was elevated in DSCs after the stimulation with TNF-α and IL1β. The bars indicate the ratio of the intensity of NOD2 protein to that of β-actin. Error bars depict the standard error of the mean. Data represent mean of fold change ±SEM. * P<0.05, ** P<0.01, compared with the vehicle control.

Since MDP-mediated NOD2 expression activated the secretion of pro-inflammatory cytokines in DSCs, we next wanted to determine if cytokines would have an effect on the expression of NOD2 as part of a positive feedback loop. To define the contribution of cytokines to NOD2 expression in DSCs, we stimulated normal DSCs with the inflammatory cytokines IL-1β, TNF-α, IFN-γ (a Th1 Type cytokine), and IL-17A (an IL-17 type cytokine). These four cytokines are commonly secreted at the maternal-fetal interface during pathogen infection. As shown in Figure 4A and 4C, IL-1β induced the expression of NOD2 mRNA and protein in a dose dependent manner. Compared with the vehicle control (0 ng/ml), TNF-α (1, 10, and 100 ng/ml) induced normal DSCs to express significantly higher NOD2 protein levels. However, TNF-α treatment did not affect the mRNA expression. Both IL-1β and TNF-α induced significantly higher NOD2 protein expression in normal DSCs.

### MDP stimulation up-regulated the apoptosis of DSCs

**Figure 5:**
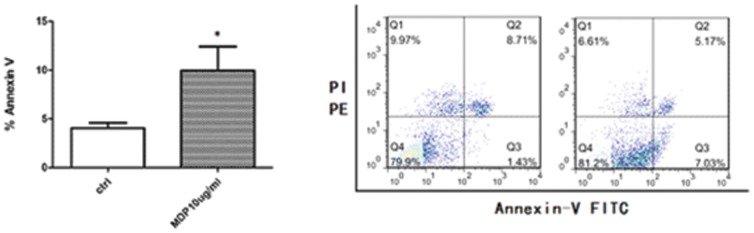
Apoptosis of DSCs pretreated with MDP (10 µg/ml) for 48 h or the control vehicle was compared by flow cytometry. Cells stained by AnnexinV but not PI (AnnexinV+/PI −) were considered to be apoptotic cells, whereas those positive for both markers (AnnexinV+/PI+) were necrotic or advanced apoptotic cells. Error bars depict the standard error of the mean. Data represents the mean of fold change ±SEM. * P<0.05, ** P<0.01.

A higher rate of apoptosis has been considered as one of the mechanisms for early abortion [32]. Thus, we tested whether the NOD2 expression in the primary DSCs was associated with increased apoptosis by flow-cytometry. We found that MDP (10 µg/ml) stimulation of the first-trimester DSCs induced significantly higher rate of apoptosis (Fig. 5) in a dose-dependent manner (data not shown).

## Discussion

Within the decidua, there are many immune cells that can mount an immune response against pathogen infection [33]. However, DSCs are non-immune cells that are the predominant cell type at the maternal–fetal interface, comprising 75% of decidual tissue. In addition to decidual immune cells, the non-immune DSCs have been shown to express PRRs, including TLRs and NOD-likes receptors (NLR). Although other reports have shown that DSCs express TLR-2 and TLR-4 proteins during the first trimester [28], [29], whether DSCs expressed NOD2 was unknown. To our knowledge this is the first report demonstrating that NOD2 was expressed in the decidua during the first trimester and that it functions as a key regulator of cytokine secretion. Thus, the data is consistent with other reports of PRR expression. These results suggest that non-immune DSCs at the maternal–fetal interface have the potential to recognize and respond to pathogens and may also play a role in the infection associated with adverse pregnancy outcome.

NOD2 expression also has been observed in first trimester placenta, specifically the trophoblast cells [22], and in endometrial epithelial cells [23]. The immunohistochemical localization of NOD2 indicated its wide expression in deciduas, mainly in the decidual granular epithelium but also prevalently in stromal cells (DSCs), the predominant cellular component of the deciduas. While trophoblasts, endometrial epithelial cells, and DSCs express NOD2, MDP stimulation of the three cell types induced overlapping but not identical subsets of cytokines. For example, MDP stimulation of endometrial epithelial cells significantly increased expression of IL-8 and TNF-α [23] whereas MDP treatment did not significantly increase TNF-α secretion from our DSCs from normal first term pregnant women. MDP stimulation of trophoblasts from the first trimester significantly augmented GRO-α, IL-6, IL-8, and MCP-1 expression levels [23] and MDP stimulation of our normal DSCs from first trimester pregnancies also significantly increased MCP-1 expression levels. Taken together, these data suggest that NOD2 is functional in DSCs, trophoblasts [22], and endometrial epithelial cells [23], and its stimulation with MDP differentially induces various cytokines in the Th1 paradigm [22], [23]. The production of overlapping cytokine subsets likely aids the tight regulation of Th1 responses necessary to maintain the maternal tolerance towards the fetus. Interestingly, MDP stimulation of Nod2 in a murine macrophage cell line releases its tight chaperone, heat shock protein 90, and degrades the activated Nod2 protein, which blocks Nod2's continual activation [34]. If MDP stimulation also downregulates expression of NOD2 in human trophoblasts and DSCs, the short burst of NOD2 activation can contribute to the highly regulated temporal and spatial expression of chemokines/cytokines in the decidua and placenta.

Increased expression of NOD2 mRNA in response to cytokine IL-1β has been confirmed in endometrial epithelial cells [22] and human umbilical vascular endothelial cells [35]. Also, TNF-α has been reported to elevate NOD2 expression in mouse macrophage RAW264.7 cells [36], in four epithelial cell lines and in primary colonic epithelial cells [37]. In our study, MDP-mediated NOD2 expression activated secretion of cytokines, IL-1β and MCP-1, and in turn IL-1β and TNF-α elevated the expression of NOD2 in DSCs. These results suggest that during pathogen infection, elevated cytokine secretion creates a positive feedback loop that increases the expression of NOD2, which may in turn augment inflammation at the maternal-fetal interface.

During pregnancy, pathogen infection can inadvertently lead to excessive inflammation or apoptosis at the maternal-fetal interface [38]. Costello et al. demonstrated that ectopic expression of NOD2 in a first trimester trophoblast cell line, H8, produced higher levels of cytokines than with vehicle treatment alone [22], suggesting that intracellular NOD2 expression levels affect baseline production of cytokines. NOD2 expression may be crucial for the balance of chemokine and cytokine signaling at the maternal-fetal interface that is essential during implantation and placentation [39]–[42].

It has been shown that higher apoptosis in the chorionic villi, erythroblasts, endothelium of capillaries, monocytes, and promonocytes is associated with spontaneous abortion [32]. In this study, we found MDP stimulation of NOD2 in DSCs significantly increased their apoptotic rate, which may be a contributing factor in spontaneous abortion [43]–[45]. However, further investigation is required to clarify the effect of MDP-induced apoptosis in DSCs on pregnancy outcome. Both NOD2-induced inflammation and apoptosis at the maternal-fetal interface may be contributing to adverse pregnancy outcome. Examination of the mechanism underlying NOD2 regulation at the maternal-fetal interface as well as NOD2 downstream signaling pathways will elucidate the role of NOD2 in pregnancy. Future studies should compare the effect of MDP-induced NOD2 expression on cytokine and chemokine secretion profiles in DSCs between patients with normal pregnancies and those with spontaneous abortions.

In summary, we have demonstrated that DSCs isolated from early pregnancies express the cytoplasmic pattern recognition receptor, NOD2. MDP significantly elevated the expression of NOD2 in DSCs. In addition, MDP activation of NOD2 significantly increased IL-1β and MCP-1 cytokine expression in a dose dependent manner but had no effect on IL-12 expression. IL-1β and TNF-α also significantly increased the expression of NOD2 itself, suggesting the presence of a positive feedback loop. Moreover, MDP stimulation up-regulated DSC apoptosis. These findings suggest that DSCs may play an important role in protecting the embryo and prevent infections in the maternal-fetal interface.
